# Preparation of Mercurial Ointment

**Published:** 1842-11-05

**Authors:** 


*Preparation of Mercurial Ointment.*—Mr. Redwood brought before the notice of the Pharmaceutical Society some lard prepared in a peculiar manner, for the more speedy oxydation of mercury. The process he adopts is as follows:—The lard is melted in an earthen pipkin, and, while in a fluid state, poured in a thin stream, from some height, into a vessel containing a considerable quantity of cold water. The lard diffuses itself in a thin stratum over the surface of the water; it is now to be collected and placed in a coarse hair sieve, and the top of the sieve to be covered with paper, to preserve it from the dust. In this state it should be kept in a dry apartment, exposed to the action of the air for two or three months, at the expiration of which time it will be found to have acquired the property of readily combining with a very large proportion of mercury. This process was first suggested by M. Dorly, a French chemist. Mr. Redwood then exhibited some lard thus prepared, and demonstrated to the society its advantages in combining with the metal with less labor and loss of time than is usually incurred.—*Pharmaceutical Journal.*

